# Chronic Exertional Compartment Syndrome of the Forearm in an Elite Rower

**DOI:** 10.1155/2011/497854

**Published:** 2012-01-26

**Authors:** S. O'hEireamhoin, J. F. Baker, M. Neligan

**Affiliations:** ^1^Department of Orthopaedics, AMNCH, Tallaght, Dublin 24, Ireland; ^2^Sports Surgery Clinic, Santry Demesne, Dublin 9, Ireland; ^3^Department of Trauma and Orthopaedics, AMNCH, Tallaght, Dublin 24, Ireland

## Abstract

We report a case of chronic exertional compartment syndrome (CECS) affecting the volar forearm compartment of an elite rower. CECS of the forearm is a less well recognised entity than lower limb CECS. We describe a typical history and detail a potential treatment.

## 1. Introduction

While acute compartment syndrome, often secondary to trauma, is a well-known entity, chronic exertional compartment syndrome (CECS) is a less well-recognised diagnosis. As with the acute form, CECS can affect any of the large fascial compartments but is more recognised in the lower limb [1, 2]. CECS of the forearm has previously been reported in motocross riders and kayakers [3–5]. We present a case of CECS of the forearm occurring in an elite female rower.

## 2. Case Report

A 20-year-old female presented complaining of recurrent right forearm pain for the preceding three months. The pain was felt predominantly in the volar aspect of the forearm running from the wrist to the elbow and was associated with parasthesia of the entire hand. Onset of the pain was always after exertion—she was a competitive rower and typically would experience the pain after intense rowing. She also noted slight swelling and a flexion contracture with ulnar deviation of the wrist. Following cessation of activity, the pain would subside over approximately 15 minutes and sensation return to normal. She had no medical history of note and no prior trauma to the upper limb.

**Figure 1:**
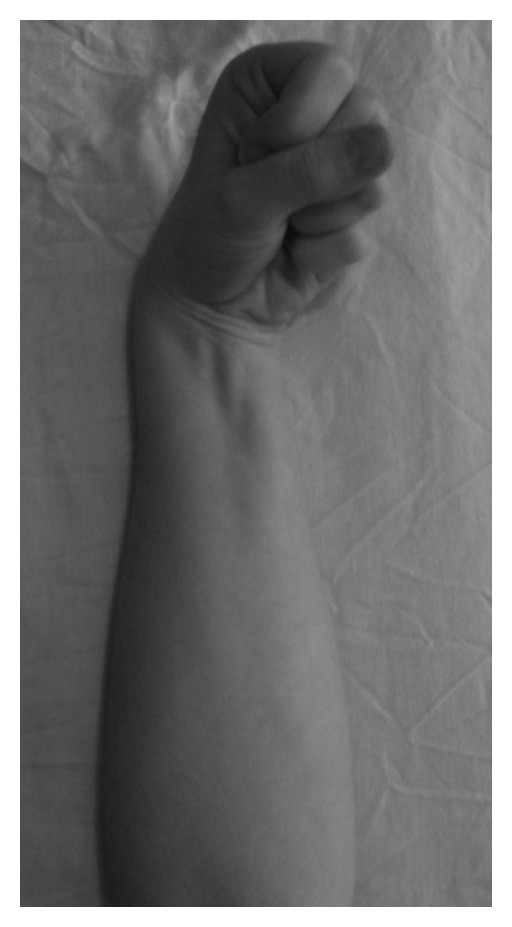
Position of the affected limb.

On examination, the upper limb appeared of normal bulk compared to the contralateral side. Assessment of tone, power, and sensation of the right upper limb was normal. Radial and ulnar pulses were palpable at the wrist. There was no objective evidence of Thoracic Outlet Syndrome and cervical spine movements were normal. After using an indoor rowing machine, the symptoms were reproduced, the volar aspect of the right forearm tense and the wrist held in a flexed and ulnar deviated disposition (Figure 1)—attempts to manually extend the wrist and digits was painful. Symptoms subsided gradually on cessation of rowing disappeared completely by 15 minutes.

Plain radiographs of the elbow to the wrist were normal. Nerve conduction studies showed a borderline carpal tunnel lesion but ulnar nerve function was normal both before and after provocation with exercise. Electromyography of the forearm musculature did not show any underlying myotonic or myopathic disorder. Compartment pressure testing of the volar compartments confirmed a raised resting pressure of 20 mmHg. A diagnosis of chronic exertional compartment syndrome of the flexor aspect of the forearm was made and surgical decompression offered.

**Figure 2:**
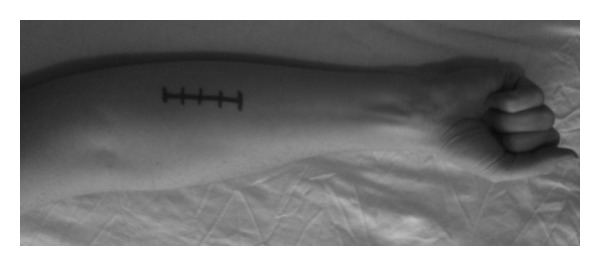
An incision was made just ulnar to the midline on the volar aspect of the forearm.

A flexor compartment release was performed of the volar compartments via an incision ulnar to the midline on the volar aspect of the forearm (Figure 2). A release was made from 2 cm distal to the elbow proximally to the musculotendinous junction distally. One month following surgery, she returned to competitive rowing with no ill effects or recurrence of symptoms.

## 3. Discussion

We have presented a case of chronic exertional compartment syndrome (CECS) occurring in a female rower. We believe this particular diagnosis is underreported but has been noted previously in motorcross riders and kayakers [5, 6]. The predisposing action in this case appears to be prolonged isometric flexion of the wrist and fingers with associated flexion of the elbow. In this case, the precipitating action was that of repetitive wrist flexion in an ulnarward direction as could be anticipated when pulling oars toward ones torso.

The history in this case was highly suggestive as was the provocative testing. Confirmation of the diagnosis obtained with compartment pressure testing and this remains the gold standard for diagnosis. Pedowitz et al. have reported that a preexercise compartment pressure of 15 mmHg or greater, a one-minute postexercise pressure of 30 mmHg or greater, or a pressure of 20 mmHg or greater five minutes postexercise are values suggestive of CECS [7]. Others have noted the contribution of resting compartment pressures (abnormal above 15 mmHg), but put an equal emphasis on the delayed return to normal [8]. However, most studies have focussed on the lower limb and there is no real consensus with regards diagnosis of this condition in the upper limb. More recently MRI findings have been described in exertional compartment syndromes of the forearm and may represent a future alternative to formal compartment pressure testing [4, 5].

Treatment of CECS may be conservative with avoidance of aggravating activity. Fasciotomy offers a treatment option in resistant cases or in those who are unable to sufficiently modify their activity. In this case, only the volar compartment was decompressed and this leads to satisfactory recovery. The patient for cosmetic reasons sought a small incision and indeed this approach can be adopted with good clinical outcomes. A larger series of 16 patients undergoing minimally invasive fasciotomies in the forearm has been reported with successful clinical outcomes [3]. The authors highlight the potential benefit of using a minimally invasive approach of a quicker recovery time.

In conclusion, exertional compartment syndrome of the forearm needs to be considered in the differential diagnosis for patient presenting with forearm or wrist pain. Although the history can be highly suggestive of the diagnosis, compartment pressure can be used to aide diagnosis. Fasciotomy can be performed via small incisions providing both cosmetically and clinically acceptable outcomes.
